# Assessment of parental knowledge on childhood developmental milestones and associated factors among parents of children under 5 years in Addis Ababa, Ethiopia

**DOI:** 10.1097/MD.0000000000051035

**Published:** 2026-10-09

**Authors:** Suleyman Mohammed Arage, Mequanente Dagnaw, Meera Indracanti, Rahul Pothapragada, Dinesh Govindraj, Chandrakala Gunturu, Halima Kasim, Basmah Abdulaziz Albabtain, Tabarak Malik

**Affiliations:** a Department of Public Health, College of Medicine and Health Science, Werabe University, Worabe, Ethiopia; b Department of Health System and Policy, Institute of Public Health, College of Medicine and Health Science, University of Gondar, Gondar, Ethiopia; c Department of Epidemiology and Biostatistics, Institute of Public Health, University of Gondar, Gondar, Ethiopia; d Department of Medical Biotechnology, Institute of Biotechnology, University of Gondar, Gondar, Ethiopia; e Department of Medical Biotechnology, School of Allied and Healthcare Sciences, Malla Reddy University, Hyderabad, Telangana, India; f Department of Computing Technologies, SRM Institute of Science & Technology, Kattankulathur, Chennai, India; g Microl Remedies, Kukatpally, Hyderabad, Telangana, India; h Department of Public Health, Ayer Tena Health Science and Business College, Addis Ababa, Ethiopia; i Department of Pharmacy Practice, College of Pharmacy, Princess Nourah bint Abdulrahman University, Riyadh, Saudi Arabia; j Division of Research and Development, Lovely Professional University, Phagwara, India.

**Keywords:** Addis Ababa, developmental milestone, knowledge, parents

## Abstract

Parents’ knowledge of developmental milestones helps promote healthy development and appropriate expectations. Limited knowledge may contribute to neglect, abuse, and delayed recognition of developmental problems. Despite efforts to prevent developmental delays in Ethiopia, evidence on parental knowledge of developmental milestones remains limited. Therefore, this study aimed to assess parental knowledge of child developmental milestones and associated factors among parents of children under 5 years in Addis Ababa, Ethiopia. A community-based cross-sectional study design was conducted among 3 sub-cities from July 15 to August 30, 2024, among 423 parents selected by a systematic random sampling technique. The data was collected by using interviewer-administered questionnaires. The descriptive part was presented using narration, tables, and figures. A bivariable logistic regression statistical analysis was used to identify potential candidate variables (*P* < .25) for multivariable logistic regression analysis to control potential confounding variables; the regression result was presented using adjusted odds ratio (AOR) with 95% confidence interval (CI) and *P* value <.05 as level of significance. Overall, 30%, 95% CI: (25.6–34.3%) of parents had good knowledge about childhood developmental milestones. Sex (being female) AOR = 3.54, 95% CI:(1.30–9.67), family size (≥5) AOR = 2.73, 95% CI: (1.45–5.14), and source of information (social media) AOR = 2.34, 95% CI: (1.88–3.12) were factors that had a significant association with parental knowledge of childhood developmental milestones. Overall, fewer than one-third of parents had good knowledge of childhood developmental milestones. Parental knowledge was significantly associated with sex, family size, and source of information. These findings highlight the need for targeted educational interventions to improve parents’ knowledge of childhood developmental milestones.

## 1. Introduction

Parenting knowledge refers to parents’ understanding of child developmental norms and milestones, developmental processes, and appropriate caregiving practices.^[1]^ Developmental milestones are functional skills or behaviors that most children achieve within a predictable age range and serve as important indicators of healthy physical, cognitive, language, emotional, and social development.^[2–4]^ Timely recognition of developmental delays enables early intervention, which can substantially improve children’s developmental outcomes and quality of life.^[3,4]^

Despite global efforts to promote early childhood development, developmental delays remain a major public health concern. According to the United Nations Children’s Fund, approximately 36.8% of children aged 3 to 4 years in low- and middle-income countries fail to achieve expected cognitive and socio-emotional developmental milestones.^[5]^ Similarly, the World Health Organization estimates that developmental disabilities affect a considerable proportion of children under 5 years of age, while psychomotor and language disorders are estimated to occur in 12 to 16% of young children.^[6,7]^ Although routine developmental screening improves early identification and referral for intervention, many children with developmental delays remain undetected until school age, reducing opportunities for timely treatment.^[8–10]^

Parents play a central role in recognizing developmental milestones and seeking appropriate care. However, studies conducted in different countries have reported considerable variation in parental knowledge of child development. These differences are likely attributable to variations in parental educational attainment, access to healthcare and developmental information, cultural beliefs and child-rearing practices, socioeconomic conditions, and the measurement tools used to assess knowledge.^[11,12]^ Limited parental knowledge has been associated with unrealistic developmental expectations, inappropriate parenting practices, delayed healthcare seeking, and poorer developmental outcomes among children.^[11–14]^

In Ethiopia, several initiatives, including nutrition programs, salt iodization, and early childhood development strategies, have been implemented to improve child health and development.^[3]^ Nevertheless, evidence suggests that many parents have limited awareness of normal developmental milestones, particularly in cognitive, language, and socio-emotional development. Cultural context also influences how parents perceive and respond to children’s developmental progress, highlighting the importance of assessing parental knowledge using locally relevant approaches. Despite the importance of parental knowledge for early detection and intervention, evidence on its magnitude and associated factors remains limited in Ethiopia, particularly in Addis Ababa. Therefore, this study aimed to assess parental knowledge of childhood developmental milestones and associated factors among parents of children under 5 years of age in Addis Ababa, Ethiopia.

## 2. Methods

### 2.1. Study area and period

The study was conducted in Addis Ababa, the capital city of Ethiopia. Addis Ababa is the largest city with a total population of 3,384,569 according to the 2007 census. This city has shown a robust annual growth rate, and population counts as of 2017 are growing closer to 4 million, and holds 527 square kilometers of area in Ethiopia, with a density of about 5165 individuals per square kilometer.^[15]^ Administratively, the city was divided into 11 sub-cities and over above120 woredas. There were 5 hospitals under the Ministry of Health, 6 Hospitals under the Addis Ababa Health Bureau, 1 Hospital under the Ministry of Defense, 1 Hospital under the Police Force, and around 40 Private Hospitals. The city also had more than 102 functional governmental health centers and more than 760 private clinics in 11 sub-cities.^[16]^ The study was confined to Kolfe Keranio sub-city, Lideta sub-city, and Addis Ketema sub-city. The study was conducted from July 15 to August 30, 2024.

### 2.2. A study design

A community-based cross-sectional study design was implemented.

### 2.3. Population

#### 2.3.1. Source population

All parents with households aged <5 years in the selected sub-city of Addis Ababa, Ethiopia.

#### 2.3.2. Study population

Randomly selected parents with children aged <5 years in the selected sub-city during the study period in Addis Ababa, Ethiopia.

### 2.4. Inclusion and exclusion criteria

*Inclusion criteria:* Parents who had at least one child under the age of 5 during the study period were included.

*Exclusion criteria:* Parents whose children were diagnosed with developmental delay were excluded, as they are already aware of delayed milestones.

### 2.5. Sample size and sampling technique

#### 2.5.1. Sample size determination

The sample size was calculated using a single proportion formula by assuming a 50% proportion since no study has been done on parental knowledge of childhood developmental milestones. Other assumptions made during the sample size calculation are a 5% marginal error (d) and a confidence interval (CI) of 95% (*Z* = 1.96). Based on these assumptions, the sample size was calculated as follows: In the calculation of sample size, the following points will be considered: p = Parental Knowledge on childhood developmental milestones, d = the margin of sampling error tolerated or degree of precision = 5%, *z*α/2 is the standard normal variable at 1−α% confidence level = 1.96, α = 5%, and n = minimum sample size.

Based on the above assumption, n = 384. By adding a 10 % nonrespondent rate, the final sample size was 423.

#### 2.5.2. Sampling technique/procedure

There were eleven sub-cities in Addis Ababa. Including 30% of Addis Ababa’s sub-cities, 3 sub-cities (Kolfe Keranio sub-city, Lideta sub-city, and Addis Ketema sub-city) were included by a systematic random sampling technique. Again, from the woreda found in each sub-city, by including 30% of the sub-cities’ woreda, 9 woreda were included by lottery methods. The total sample size calculated for the study was distributed to the selected woreda using proportional allocation. Proportional allocation was done based on the number of eligible parents found in each woreda. For this allocation, a sampling fraction (=n/N) was used for all the parents proportionally, making it proportional to the number of parents. The study participants were selected using a systematic random sampling method by calculating the *k* value. To get the *k* value, divide the total number of parents found in each woreda by the sample size. Therefore, parents were interviewed at every 12th interval until the total sample size of 423 was reached.

### 2.6. Study variables

#### 2.6.1. Dependent variables

Parental knowledge of developmental milestones.

#### 2.6.2. Independent variables

*Socio-demographic characteristics:* Age of the mother, marital status, family size, education status of mother & husband, occupation of mother & her partner, number of children, family income, place of residence, and source of information.

### 2.7. Operational definition

*Parental Knowledge:* Parental knowledge of childhood developmental milestones was assessed using a structured 17-item questionnaire covering the physical, emotional, social, and cognitive developmental domains. Each correct response was assigned a score of 1 and each incorrect response a score of 0, resulting in a total knowledge score ranging from 0 to 17. In the absence of a standardized cutoff point, the sample mean knowledge score was used as the threshold to classify parental knowledge. Participants scoring above the mean were categorized as having good knowledge, whereas those scoring at or below the mean were categorized as having poor knowledge. This approach is widely used in health knowledge research to reflect the distribution of knowledge scores within the study population.^[5]^

*Developmental delay:* Refers to a child who has not gained the developmental skills expected of him or her, compared to others of the same age.

*Parents:* Fathers or mothers.

*Income categories:* The total amount of money earned by all household members from all sources (salary, business, farming, remittances, pensions, and other income) during the last month before data collection. It is categorized as follows: Low income: < 3000 Ethiopian birr (ETB), Middle income: 3000–7999 ETB/month, and High income: ≥ 8000 ETB/month.

*Merchant:* A person whose primary occupation involves buying and selling goods for profit, including retail or wholesale activities, through shops, markets, street vending, or small trading businesses, regardless of business size or registration status.

*Source of information:* Refers to the main channels through which the respondent reported receiving information about child development, health, and developmental milestones.

### 2.8. Data collection tool and procedure

#### 2.8.1. Data collection tools

The questionnaire consisted of 2 parts. Part I collected participants’ sociodemographic characteristics, including maternal age, marital status, family size, educational status of the mother and her partner, occupation of the mother and her partner, number of children, monthly family income, place of residence, and source of information. Part II assessed parental knowledge of childhood developmental milestones using a 17-item questionnaire adapted from Aldayel et al.^[17]^ The instrument included 4 domains: physical development (4 items), cognitive development (3 items), social development (5 items), and emotional development (5 items). Each correct response was scored as one point, yielding a total score ranging from 0 to 17.

Before implementation, the questionnaire was reviewed by experts in pediatrics, public health, and child development to evaluate its content relevance, clarity, and cultural appropriateness for the Ethiopian context. Based on their recommendations, minor modifications were made to improve wording while maintaining the original meaning of the items. The questionnaire was then translated from English into Amharic and back-translated into English by independent bilingual translators to ensure conceptual equivalence. A pretest was conducted on 5% of the sample outside the study area, and the questionnaire was further refined based on the pretest findings. The internal consistency of the final instrument was assessed using Cronbach alpha (≥0.90), indicating excellent reliability.

#### 2.8.2. Data collection procedures

Data were collected using interviewer-administered structured questionnaires. The study participants were invited to participate voluntarily by explaining the rationale of the study at the time of data collection. Three trained diploma midwives, blinded to study objectives, were used as data collectors to administer pretested questionnaires under the supervision of 1 BSc midwife.

### 2.9. Data quality control

To reduce the likelihood of bias, probability sampling methods were employed alongside well-defined inclusion and exclusion criteria. The data collection instrument underwent pretesting on 5% of the sample to verify its clarity, with subsequent adjustments made as necessary. Data collectors and supervisors participated in a 2-day training program covering study objectives, interviewing techniques, and ethical considerations. Daily oversight and verification of completed questionnaires were implemented. Data entry was executed through a double-entry system, and data cleaning was performed prior to analysis. During the analysis phase, multivariable logistic regression was utilized to account for potential confounders. The entire research process adhered to standardized protocols to guarantee data quality and reduce bias.

### 2.10. Data analysis

After data collection, the filled questionnaires were coded, checked for consistency and completeness, and entered into Epi Data version 3.1 statistical software. The entered data was then exported to Statistical Package for Social Sciences version 27 for further management and analysis. Before analysis, data cleaning and cross-checking were done. Both descriptive and analytical statistical procedures were performed. Descriptive analysis, such as frequencies, percentages, means, and medians, was computed. Bivariate binary logistic regression analysis and multivariable logistic regression analysis were used to assess the association of each independent variable with the level of knowledge and variables with a *P*-value of < .25 and < .05, respectively. Finally, the results will be presented in the form of texts, tables, and graphs.

### 2.11. Ethical clearance

Ethical approval for this community-based cross-sectional study was obtained from the Institutional Review Board of Genet and Tekba College (Reference No. Genet and Tekba College (GTC)/28/08/2024). Eligible parents or legal guardians of children under 5 years of age were approached during the data collection period, informed about the objectives and procedures of the study, and invited to participate voluntarily. Written informed consent was obtained from all participants before the interviewer-administered questionnaire was conducted. Participants were informed of their right to decline participation or withdraw from the study at any time without any consequences. To protect participants’ privacy, no personal identifiers were collected, and all data were kept confidential and used solely for research purposes. All study procedures were conducted in accordance with the ethical principles of the Declaration of Helsinki (2013 revision).

## 3. Results

### 3.1. Socio-demographic characteristics

The total number of parents who were approached for participation in the study was 423, of whom 416 agreed to be included in the study, giving a participation rate of 98.3%. Two hundred forty-two (58.5%) of study parents were found to be below the age of 30 years; only 63 (15.2%) of them were male participants, and 53 (12.8%) of them had more than 5 family members. Regarding the educational and occupational status of the mother and husband, the majority had completed primary education and were merchants by occupation. Concerning monthly income and source of information, 271 (65.5%) of them were low-paid, while television was the major source of information mentioned by the majority of the participants (Table 1).

**Table 1:** Sociodemographic characteristics of parents of children under 5 years in Addis Ababa, Ethiopia 2024.

Variables	Category	Frequency	Percent
Age	<30 yrs	242	58.5
	≥30 yrs	172	41.5
Sex	Male	63	15.2
	Female	351	84.8
Marital status	Married	347	83.8
	Divorced	55	13.3
	Widowed and other	12	2.9
Educational level	No formal education	10	2.4
	Primary	254	61.4
	Secondary	117	28.3
	College and above	33	8.0
Educational level of husband	Primary	194	46.9
	Secondary	150	36.2
	College and above	70	16.9
Current occupation	Housewife	105	25.4
	Government employee	32	7.7
	Merchants	193	46.6
	Daily laborer and others	84	20.3
Current husband occupation	Government employee	69	16.7
	Merchants	170	41.1
	Daily laborer	143	34.5
	Others	32	7.7
Family size	<5	361	87.2
	≥5	53	12.8
Monthly income	<5019 birr/months	271	65.5
	≥5019 birr/months	143	34.5
Source of information	TV	313	75.6
	Social media	8	1.9
	HPs	38	9.2
	Others	55	13.3

HPs = health care providers, TV = television.

### 3.2. Domain of parental knowledge on childhood developmental milestones

Parental knowledge was assessed using 4 domains. From the physical domain, when their child dress by them self were an item that correctly answered by majority (86%), From emotional development domain different pitches of cries was answered by 44.2%, moreover play alone for an hour item was social development domain that correctly answered by 12.6%. Furthermore, from the cognitive development domain, 63.3% know when the child counts numbers (Table 2).

**Table 2:** Domain on parental knowledge of childhood developmental milestones among parents of children under 5 years in Addis Ababa, Ethiopia, 2024.

Variables	Correct	Category	Frequency	Percent
Physical development domain				
When does the child walk	12–18 mo	Correct	332	80.2
When does the child become able to dress themselves	24–36 yrs	Correct	356	86.0
When does the child start crawling	6–12 mo	Correct	303	73.2
When does the child try to reach the objects	4–6 mo	Correct	302	72.9
Emotional development				
When does a child exert independence	12–18 mo	Correct	36	8.7
When does a child recognize others’ emotions	6–12 mo	Correct	22	5.3
When does a child make different pitches of cries	4–6 mo	Correct	183	44.2
When does a child Bond with a caregiver	4–6 mo	Correct	68	16.4
When does a child Advocate for fairness	5–6 yrs	Correct	33	8.0
Social development				
When does the child choose his friends	18–24 mo	Correct	15	3.6
When does the child agree to share his toys with other children	3–5 yrs	Correct	47	11.4
When a child chooses parallel play	18–24 mo	Correct	10	2.4
When does the child play alone for 1 hour	3–5 yrs	Correct	52	12.6
When does the child show empathy	5–6 yrs	Correct	13	3.1
Cognitive development				
When does the child count numbers	2–3 yrs	Correct	262	63.3
When does the child engage in pretend/fantasy play	12–18 mo	Correct	26	6.3
When can a child follow orders and Simple directions	12–18 mo	Correct	31	7.5

### 3.3. Parental knowledge of childhood developmental milestones

Regarding parental knowledge, out of the total participants, 124 (30%) of parents had good knowledge about childhood developmental milestones. The cognitive development domain was the most knowledgeable, with 274 (66.2%) of parents showing good knowledge, while only 104 (25.1%) of them had good knowledge of the social development domain, as shown in Figure 1.

**Figure 1.**
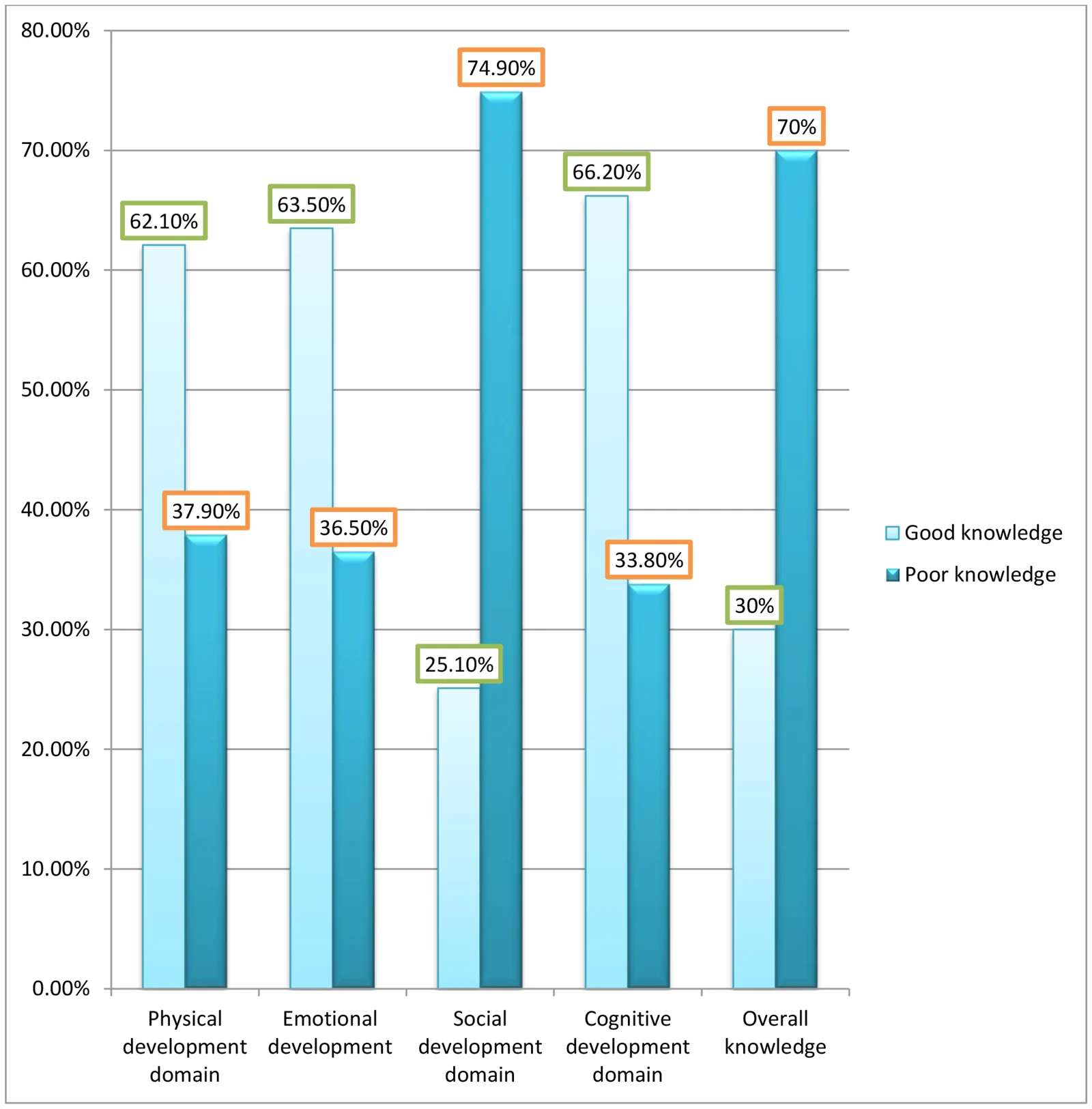
Parental knowledge of childhood developmental milestones among parents of children under 5 years in Addis Ababa, Ethiopia, 2024.

### 3.4. Factors associated with parental knowledge of childhood developmental milestones

In the bivariate binary logistic regression analysis, variables including sex, marital status, highest educational level of the respondent, husband’s highest educational level, current occupation, husband’s occupation, family size, monthly household income, and source of information met the screening criterion (*P* ≤ .25) and were considered for further analysis. In addition to statistical significance, the inclusion of these variables in the multivariable logistic regression model was guided by conceptual and empirical evidence suggesting their relevance to parental knowledge of childhood developmental milestones. Sociodemographic factors such as sex, marital status, and educational attainment influence access to health information, caregiving roles, and decision-making power. Occupational status and household income reflect socioeconomic position, which affects exposure to health education and information-seeking behavior. Family size may influence parental attention, experience, and resource allocation, while the source of information directly affects awareness and understanding of child developmental milestones. Therefore, these variables were entered into the multivariable logistic regression model to control for potential confounding and to identify independent predictors of parental knowledge. Variables with a *P*-value ≤ .05 in the multivariable model were considered to have a statistically significant association with parental knowledge of childhood developmental milestones. The Hosmer–Lemeshow goodness-of-fit test was used to assess the multivariable model fitness, and the results showed an excellent model fit (*P*-value = .94). Variance inflation factors were used to evaluate multicollinearity, and the independent variables showed no signs of multicollinearity (variance inflation factor < 1.16).

The odds of parental knowledge of childhood developmental milestones are 3.54 times higher among female parents compared with male parents, with adjusted odds ratio (AOR) = 3.54, 95% CI: (1.30–9.67).

The odds of parental knowledge of childhood developmental milestones were 2.73 times higher among parents with more than 5 family members compared with those with fewer than 5 family members (AOR = 2.73, 95% CI: [1.45–5.14]). Those parents who use social media were 2.34 times more likely to have good knowledge of childhood developmental milestones (AOR = 2.34, 95% CI: [1.88–3.12]; Table 3).

**Table 3:** The bivariable and multivariable regression on parental knowledge of childhood developmental milestones among parents of children under 5 years in Addis Ababa, Ethiopia, 2024.

Variable	Category	COR (95% CI)	*P*-value	AOR (95% CI)	*P*-value
Age	≥30 years (Ref)	1		1	
	<30 years	2.23 (1.45–3.59)	<.001	2.29 (0.91–3.64)	.087
Sex	Male (Ref)	1		1	
	**Female**	**2.24 (1.13–4.47**)	**.021**	**3.54 (1.30–9.67**)	**.014**
Marital status	Divorced/Widowed (Ref)	1		1	
	Married	1.95 (1.02–3.73)	.042	0.52 (0.22–1.21)	.125
Maternal education	Below secondary school (Ref)	1		1	
	Secondary and above	1.42 (0.92–2.18)	.115	1.25 (0.35–4.48)	.729
Husband’s education	Primary (Ref)	1		1	
	Secondary	3.32 (2.03–5.43)	<.001	2.12 (0.33–5.38)	.141
	College and above	3.14 (1.71–5.74)	<.001	2.92 (0.98–4.14)	.122
Current occupation	Housewife (Ref)	1		1	
	Government employee	2.47 (1.10–5.54)	.028	2.95 (0.82–10.58)	.097
	Merchant	0.70 (0.42–1.19)	.189	0.83 (0.31–2.22)	.712
	Daily laborer/others	1.03 (0.56–1.91)	.917	1.26 (0.46–3.48)	.657
Husband occupation	Government employee (Ref)	1		1	
	Merchant	1.86 (1.03–3.34)	.040	1.56 (0.81–2.61)	.094
	Daily laborer	0.18 (0.08–0.40)	<.001	0.06 (0.01–1.31)	.061
	Others	1.28 (0.53–3.08)	.579	4.07 (0.88–8.82)	.173
Family size	<5 (Ref)	1		1	
	**≥5**	**1.77 (1.08–2.91**)	**.024**	**2.73 (1.45–5.14**)	**<.001**
Monthly income	Low (Ref)	1		1	
	High	1.66 (1.08–2.56)	.022	1.26 (0.39–4.06)	.702
Source of information	Television (Ref)	1		1	
	**Social media & others**	**2.82 (1.77–4.51**)	**<.001**	**2.34 (1.88–3.12**)	**<.001**

Bold values indicate statistical significance at *P* < .05.

AOR = adjusted odds ratio, CI = confidence interval, COR = crude odds ratio, Ref = reference category.

## 4. Discussion

Overall, 124 (30%), 95% CI: (25.6–34.3%) of parents had good knowledge about childhood developmental milestones. The cognitive development domain was the most knowledgeable among most of the 274 (66.2%) parents, while only 104 (25.1%) of them had good knowledge of the social development domain. This finding is lower than the study on Parental knowledge of children’s developmental milestones in Aseer, Saudi Arabia revealed that 48% participants were found to have an acceptable level of knowledge,^[18]^ lower than from study done on Knowledge of Parents about Child Developmental Milestones in Al-Kharj City, Saudi Arabia found that overall percentages of correct answers was 49%,^[19]^ from study done A study on Saudi mothers’ knowledge and awareness of infant developmental milestones showed that mothers were highly knowledgeable about physical safety issues, including attending an infant in the tub (99%) and infant position in bed (82.3%)^[20]^ and from a study to assess knowledge about child development in caregivers attending the child psychiatry outpatient department in India indicated that 84% of caregivers were mothers. On average, caregivers were able to answer only 36% of the questions correctly.^[21]^

However higher than a study done on parents’ knowledge of childhood developmental milestones and the early identification of children with developmental delay in Jeddah, which showed that only 11% of parents had the required level of knowledge. The motor domain had the highest level of accuracy, followed closely by the cognitive domain. Social domains exhibited lower levels of accuracy,^[22]^ from another study on parental knowledge of children’s developmental milestones in Riyadh, Saudi Arabia, indicated that Most respondents showed 20% of knowledge in all 4 domains.^[23]^ From study conducted in India showed that the level of maternal knowledge about child developmental milestones in Kanchipuram is relatively low.^[24]^

Regarding factors associated with parental knowledge of childhood developmental milestones, sex, family size, and source of information were significant factors. The odds of parental knowledge of childhood developmental milestones are 3.54 times higher among mothers compared with fathers. This is because mothers rarely have responsibility rendered due to cultural context, so their attachment with children is frequent, and they experience good knowledge. This finding is supported by a study done in Jeddah, which identified educational level and gender among the potential factors that may influence parents’ level of knowledge, educational level, and gender, a significant association with a positive trend towards the highest level of education, while women exhibited a more appropriate level of knowledge,^[22]^ Study in Riyadh, Saudi Arabia, identified the knowledge gaps of both parents and found that mothers are twice as knowledgeable as fathers^[23]^ and a study done in Aseer, Saudi Arabia, found that mothers achieved acceptable knowledge of developmental milestones better than fathers, and better knowledge was found in less crowded houses.^[18]^

The odds of parental knowledge of childhood developmental milestones were 2.73 times higher among parents with more than 5 family members compared with those with fewer family members. This is because parents who had more children were more likely to experience the stage at which the child attained those developmental milestones.

More over those parents who use social media were 2.34 times more likely to have good knowledge on child hood developmental milestone than those use television. This might be due to currently most of parents use social media such as tiktok, youtube and facebook as source of information.

## 5. Conclusion

Less than one-third of parents showed adequate understanding of childhood developmental milestones, according to one survey. Parental knowledge was found to be statistically correlated with parental sex, family size, and information source; however, these correlations should be interpreted cautiously because the study’s cross-sectional methodology precludes causal inference. The results point to parental knowledge gaps that might need to be addressed. Parental knowledge may be improved by bolstering parental education programs in primary healthcare settings, integrating developmental milestone conversations into regular child health visits, and encouraging community-based awareness campaigns, especially those that make use of easily accessible digital and mass media platforms. However, these suggestions should be regarded as exploratory and evaluated in light of other statistical constraints and possible residual confounding, highlighting the need for additional longitudinal and interventional research.

## 6. Strengths and limitations

### 6.1. Strengths

This study used a cross-sectional design, which allowed for efficient assessment of parental knowledge on childhood developmental milestones and identification of associated factors. It enabled the collection of data from a large number of parents within a limited time and resource frame. The findings provide essential baseline information for planning child development education programs.

### 6.2. Limitations

The cross-sectional design of the study precludes establishing causal relationships between parental knowledge and the associated factors. In addition, the use of self-reported data may have introduced recall and social desirability biases. Although the questionnaire was adapted and pretested, some measurement bias may still have occurred due to differences in cultural interpretation of developmental milestones. Furthermore, as the study was conducted in Addis Ababa, the findings may not be generalizable to parents in rural areas or other regions of Ethiopia.

## Acknowledgments

The authors sincerely thank the healthcare professionals at the hospital for their generous support and invaluable assistance in data collection and chart retrieval. They also extend their appreciation to the data collectors and supervisors for their contributions. Furthermore, the authors gratefully acknowledge the support provided by the Princess Nourah bint Abdulrahman University Researchers Supporting Project (PNURSP2026R816), Princess Nourah bint Abdulrahman University, Riyadh, Saudi Arabia.

## Author contributions

**Conceptualization:** Mequanente Dagnaw.

**Data curation:** Suleyman Mohammed Arage, Mequanente Dagnaw, Meera Indracanti, Rahul Pothapragada, Chandrakala Gunturu, Halima Kasim, Basmah Abdulaziz Albabtain, Tabarak Malik.

**Formal analysis:** Halima Kasim.

**Investigation:** Dinesh Govindraj.

**Validation:** Dinesh Govindraj.
